# Cross-sectional study on surgical site infections and adherence to intraoperative sterile protocols

**DOI:** 10.6026/973206300211623

**Published:** 2025-06-30

**Authors:** Naveenkumar Viswanathan, Namratha Sivaprasad Dasamantha, Kaushik Nattamai Rameshbabu, Ajeet Saoji, Naveen Javari Thirumalapura Javarashetty, Keerthika Muniasamy

**Affiliations:** 1 Department of General Surgery, Prince Charles Hospital, CWM TAF Morgannwg University Health Board, Wales, UK; 2 Department of Nephrology, Nanavati Max Superspeciality Hospital, Mumbai, Maharashtra, India; 3 Department Surgery, Madras Medical College, Chennai, Tamil Nadu, India; 4 Department of Community Medicine, N. K. P. Salve Institute Of Medical Sciences & Research Centre And Lata Mangeshkar Hospital, Digdoh, Nagpur, Maharashtra, India; 5 Department of General Surgery, JP Hospitals Hyderabad, Telangana, India; 6 Department of Surgery, Madras Medical College, Chennai, Tamil Nadu, India

**Keywords:** Surgical site infections, intraoperative protocols, infection control, sterility, surgical outcomes, cross-sectional study

## Abstract

Despite advancements in surgical care, surgical site infections (SSIs) continue to contribute significantly to postoperative morbidity. Hence, we evaluated 150 surgical cases over six months at a tertiary care center to assess SSI prevalence and intraoperative sterile practice adherence. An overall SSI rate of 12.7% was noted, predominantly in gastrointestinal and emergency surgeries. Key contributing factors included prolonged operation time, inadequate sterilization, and poor adherence to sterile protocols. The findings underscore the need for stringent infection control practices to reduce SSIs and improve surgical outcomes.

## Background:

Surgical site infections (SSIs) are one of the most prevalent health care-associated infections and are a major cause of patient morbidity, hospital stay, and other health care expenses. Despite advances in surgical technique and aseptic practice, SSIs remain a significant problem in developing and developed nations [1]. The SSIs are responsible for up to 20% of all healthcare-associated infections in surgical patients. This varies with the surgical procedure, patient factors and compliance with sterile protocols [2]. The etiology of SSIs is multifactorial and is a result of the interaction of a number of patient-related factors, surgical technique, and perioperative environmental factors [3]. Risk factors are high with long operative time, surgical wound contamination and failure to comply with sterile protocols during surgery. Emergency operations and gastrointestinal operations are particularly at risk due to greater microbial exposure and poor sterile barriers [4]. This cross-sectional survey sought to identify the incidence of SSIs and assess adherence to intraoperative sterile protocols in a tertiary care institution [5]. Therefore, it is of interest to identify infection control practice gaps areas and correlate them with SSI incidence to facilitate practical recommendations for improving surgical outcomes and reducing avoidable complications.

## Materials and Methods:

This cross-sectional study was conducted over a period of six months in a tertiary care hospital to assess the prevalence of surgical site infections (SSIs) and adherence to intraoperative sterile protocols. In all, 150 surgical cases were included; the range covered elective and emergency procedures. The patients who underwent surgery during the study period were enrolled if aged 18 years and above; those with a pre-existing infection or undergoing a procedure outside the sterile operating environment were excluded. Information was gathered directly by observing the surgeries and from patients' files with regard to the age, sex, type of operation, type of wound, operation time, and compliance with the intraoperative aseptic procedure. Parameters used included the usage of PPE, proper hand hygiene, sterilization of the surgical instruments, and adherence to antiseptic skin preparation. Postoperative follow-up was performed to diagnose SSIs, and these were those infections occurring in the first 30 days post-surgery using the CDC standards. Laboratory experiments, including the culture and sensitivity of the wounds, were conducted for suspected cases of infection. Statistical analysis to determine factors influencing SSIs were done with the level of significance set at p < 0.05.

## Results:

Table 1 (see PDF) highlights the association between operative time and SSIs, showing that procedures lasting more than 120 minutes had significantly higher SSI rates (25%) compared to those lasting 120 minutes or less (5.6%). Table 2 (see PDF) compares SSI rates between elective and emergency surgeries, with emergency procedures showing a significantly higher SSI rate (22.5%) compared to elective surgeries (8.3%). Table 3 (see PDF) depicts the distribution of SSIs by surgical specialty, with gastrointestinal surgeries accounting for the majority of cases (55%), followed by orthopedic (20%) and obstetrics and gynecology procedures (15%). Table 4 (see PDF) demonstrates the impact of adherence to sterile protocols on SSI rates, showing that poor adherence was associated with significantly higher rates of SSIs (36.7%) compared to cases with proper adherence (6.7%). Table 5 (see PDF) highlights SSI rates based on wound classification, showing that contaminated and dirty wounds had the highest rates of infection (25% and 35%, respectively) and whereas clean wounds had the lowest rate (2.5%). Table 6 (see PDF) shows the distribution of SSIs by age group, indicating that patients above 60 years had the highest SSI rate (20%) compared to younger age groups. Table 7 (see PDF) compares SSI incidence by gender, revealing slightly higher rates in males (13.3%) than in females (12.0%). Table 8 (see PDF) depicts the microbial profile of SSIs, showing that gram-negative bacteria, particularly *Escherichia coli* (35%), were the most common pathogens. Table 9 (see PDF) summarizes the antibiotic resistance patterns, revealing a high resistance rate to beta-lactam antibiotics (50%), followed by aminoglycosides (30%) and fluoroquinolones (20%). Table 10 (see PDF) shows the length of hospital stay for SSI cases, demonstrating that patients with SSIs had significantly longer hospital stays (average 12 days) compared to non-SSI cases (average 5 days). Table 1 (see PDF) illustrates the association between operative time and SSIs, showing a significantly higher infection rate (25%) in procedures lasting over 120 minutes compared to those lasting 120 minutes or less (5.6%). Table 2 (see PDF) compares SSI rates in elective versus emergency surgeries, revealing higher rates in emergency procedures (22.5%) than in elective ones (8.3%). Table 3 (see PDF) depicts the distribution of SSIs by surgical specialty, with gastrointestinal surgeries accounting for the highest percentage (55%). Table 4 (see PDF) demonstrates the impact of adherence to sterile protocols, where non-adherence resulted in a significantly higher SSI rate (36.7%) compared to cases with proper protocol adherence (6.7%). Table 5 (see PDF) highlights the role of wound classification, showing that contaminated and dirty wounds had the highest SSI rates (25% and 35%, respectively). Table 6 (see PDF) presents the distribution of SSIs by age group, with patients above 60 years showing the highest rates (20%). Table 7 (see PDF) Incidence of SSIs by Gender Males: 13.3%, Females: 12.0%. The microbial profile for SSIs in Table 8 (see PDF) identified gram-negative organisms such as *Escherichia coli* and *Klebsiella spp.* to be most frequently involved pathogens. The sum-up for resistance pattern in antibiotic therapy in Table 9 (see PDF) reports 50% resistance rate in beta-lactams, demanding updating antimicrobial protocol. Finally, Table 10 (see PDF) presents the length of stay in the hospital, and the SSI patients had significantly longer stays compared to the non-SSI cases (12 days for SSI and 5 days for non-SSI). This summary points out the operative and patient factors, adherence to sterile protocols, and microbial characteristics as influencers of SSI rates and actionable insights into infection prevention and control strategies.

## Discussion:

This study highlighted the significant burden of surgical site infections and how intraoperative sterile protocols play a critical role in reducing their incidence [6]. With an overall SSI rate of 12.7%, the findings are consistent with reported global rates, emphasizing the need for stringent infection control measures. Prolonged operative time, emergency surgeries and poor adherence to sterile protocols emerged as key contributors to increased SSI rates [7]. For example, procedures exceeding 120 minutes had an SSI rate of 25%, while non-compliance with sterile protocols resulted in a 36.7% SSI rate. These findings underscore the importance of optimizing surgical practices to minimize preventable infections. Wound classification significantly influenced SSI rates, with contaminated and dirty wounds showing the highest rates of infection (25% and 35%, respectively) [8]. Patient and procedural factors, as represented by higher SSI rates among older patients (>60 years, 20%), emergency surgeries (22.5%) and emergency procedures, can play a role in the patient's risk to develop infection [9]. The microbial profile is dominated by gram-negative organisms, such as *Escherichia coli* and *Klebsiella spp.*, to call for more targeted antibiotic prophylaxis as well as more attention to antimicrobial stewardship policies based on local resistance patterns [10]. The investigation also confirmed the impact of SSIs in the outcomes of patients. For SSI cases, patients spent an average of 12 days in hospital as compared to a non-SSI case that spent just 5 days, and there was an undeniable spike in the healthcare burden. Effective interventions, including strict adherence to sterile protocols, enhanced surgical training and multidisciplinary infection control programs, are essential to mitigate SSI risks [11]. Further future research should concentrate on the implementation and evaluation of these strategies in high-risk surgical populations to reduce the incidence of SSI further and improve postoperative outcomes.

## Conclusion:

The critical impact of surgical site infections (SSIs) on patient outcomes and healthcare systems, with a noted incidence rate of 12.7% is shown. Key contributing factors included prolonged operative time, emergency procedures and lapses in infection control measures. These findings emphasize the need for stringent aseptic practices, ongoing surgical staff training, and robust infection prevention strategies to reduce SSIs and improve postoperative outcomes.
